# Influence of structural features and feruloylation on fermentability and ability to modulate gut microbiota of arabinoxylan in *in vitro* fermentation

**DOI:** 10.3389/fmicb.2022.1113601

**Published:** 2023-01-11

**Authors:** Zhongxia Li, Huibin Zhang, Li He, Yaqin Hou, Yingjuan Che, Tian Liu, Shaobai Xiong, Xuguang Zhang, Shunjing Luo, Chengmei Liu, Tingting Chen

**Affiliations:** ^1^State Key Laboratory of Food Science and Technology, School of Food Science and Technology, Nanchang University, Nanchang, Jiangxi, China; ^2^BYHEALTH Institute of Nutrition and Health, Guangzhou, China

**Keywords:** arabinoxylan, feruloylation, carbohydrate structure, gut microbiota, *in vitro* fermentation

## Abstract

**Introduction:**

Arabinoxylan (AX) is a versatile polysaccharide that shows various effects in modulating gut microbiota and health. The influence of arabinoxylan carbohydrate structural feature and feruloylation on fermentability and the effect of modulation of gut microbiota of AX was not clear.

**Methods:**

Arabinoxylans from rice bran and corn bran (RAX and CAX), and their deferulyolated counterpart dRAX and dCAX were fermented using an *in vitro* fermentation model. Structural information was determined based on monosaccharide composition. Gas production of fermentation products, SCFAs production, pH change, and microbiota change were measured.

**Results:**

RAX and dRAX posessed lower A/X ratio compared with CAX and dCAX. The gas and total SCFAs production were lower in RAX and dRAX, and the butyrate production were higher in RAX and dRAX compared with CAX and dCAX. Butyrate production was lower at dRAX compared to RAX. On the other hand, butyrate production was higher in dCAX than in CAX. The microbiota shift were different for the four fibers.

**Discussion:**

The AXs from rice have a higher A/X ratio than the AXs from maize, suggesting more branching and a more complex side chain. The structural difference was crucial for the difference in fermentation pattern. Different *Bacteroides* species are responsible for the utilization of rice AXs and corn AXs. Although feruloylation had a minor effect on the overall fermentation pattern, it significantly affected butyrate production and alpha diversity. dRAX promoted less butyrate than RAX, which is associated with a significantly lower amount of *Faecalibacterium prausnitzi*. dCAX promoted more butyrate than CAX, which may be associated with a lower amount of *Bacteroides ovatus* and a higher amount of *Blautia* in dCAX compared to CAX. The effects of feruloylation on the fermentation pattern and the resulted microbiota shift of AX varied depending on the carbohydrate structure.

## 1. Introduction

Arabinoxylans (AXs) are an important source of dietary fiber found in various cereals such as wheat, corn, rice, sorghum and others, which possess various biological activities such as boosting immunity and antioxidant effects, strengthening the intestinal epithelial barrier, relieving constipation, and improving lipid and glucose metabolism (Comino et al., 2016; Kim et al., 2017; Wang et al., 2020). Arabinoxylans consist of a-1,4-xylan polymers that are predominantly substituted with arabinose side chains (Kaur et al., 2021) and cannot be degraded by mammalian enzymes (Paesani et al., 2020). The structures of AXs derived from different raw materials or extracted by different methods are not very consistent, each may have different unique structural features (Chen et al., 2019). The carbohydrate structure [different sugar composition, molecular weight (MW), type of branching, etc.] and feruloylation dominate the structures of AXs (Snelders et al., 2013), play a significant role in the ability of bacteria to degrade the polysaccharide (Wang et al., 2020).

The carbohydrate structure is the important factor of properties of AXs, which likely affects the fermentability of the gut fecal microbiota after AXs fermentation, including the fermentation rate of the gut microbiota, short-chain fatty acids (SCFAs) production, and the abundance and composition of the gut microbiota (Damen et al., 2011; Kaur et al., 2011; Rumpagaporn et al., 2015; Xu et al., 2022). Arabinoxylans with less branch or lower ratio of arabinose to xylose (A/X) are more easily fermented than highly branched or substituted AXs (Pollet et al., 2012; Mendis et al., 2016; Tiwari et al., 2019). Meanwhile, compared AXs with lower average degree of polymerization, the higher average degree of polymerization of AXs promoted the growth of probiotics such as *Roseburia* and *Eubacterium rectale* (*Agathobacter rectalis*), and increase in butyrate levels in the caeca of the rats (Damen et al., 2011).

Feruloylation is the binding of ferulic acid (FA) to arabinoxylan. Ferulic acid is the major bound polyphenol of AXs known for its health-promoting biological effects, such as antioxidant activity, anti-cancer activity, and cardiovascular disease, to name a few (Ghosh et al., 2017; Johnson et al., 2020). The bound FA content of AXs is related to the extraction method applied. AXs can be extracted usually using alkali and enzymatic treatments. Enzymatic treatment produced the hydrolytic degradation products of AXs (AXOS) with lower molecule weight and higher bound FA content than the AXs with alkali treated (Chen et al., 2019). The bound FA could delay the overall degradation rate of AXOS during fermentation, promote the production of SCFAs and the growth of beneficial bacteria (Snelders et al., 2014; Gong et al., 2019), while the effect of bound FA on the ability of AXs to affect the gut microbiota still unclear.

Here we assume that carbohydrate structure and feruloylation make a difference to the intestinal probiotic properties of AXs, and no relevant reports have been found. Therefore, we obtain two kinds of AXs (from rice bran and corn bran) with different carbohydrate structures but similar content of bound FA and then degrade the bound FA with feruloyl esterase to obtain two less feruloylated AXs. Investigate the carbohydrate structure and feruloylation of AXs on the intestinal probiotic properties of AX by *in vitro*.

## 2. Materials and methods

### 2.1. Materials

Defatted rice bran and corn bran powder were purchased from the Jiangxi Tianyu Oil Co., Ltd. (Jiangxi Province, China) and the native market, respectively. Human salivary α-amylase (A1031), pepsin (P7012), and pancreatin (P1750) were purchased from Sigma-Aldrich Chemical Co., Ltd. (Shanghai, China). Thermostable α-amylase (S10004), neutral protease (S10013), and 3 kDa dialysis membranes (MD3044) were purchased from Yuanye biological technology Co. (Shanghai, China). Feruloyl esterase (EC 3.1.1.73) from *Clostridium* thermocellum was purchased from Megazyme. All other chemicals and reagents were of analytical purity.

### 2.2. Extraction of arabinoxylan with different carbohydrate structures and similar content of bound ferulic acid

AXs extraction followed the procedure described previously with some modifications (Doner and Hicks, 1997). The bran was crushed to pass a 60-mesd sieve. Deionized water was added to the bran (10:1, *v*/*w*), the pH was adjusted to 7 with 2 mol/l NaOH, and then stirred for 1 h at 100°C. The starch and protein in the bran were successively removed with heat-stable α-amylase (2 g/100 g bran, hydrolysis for 1 h at 90–95°C) and neutral protease (1 g/100 g bran, hydrolysis for 6 h at 50°C and pH = 6), then the solution was boiled for 15 min to inactivate the enzymes. The reaction solution was centrifuged to obtain supernatant and residue. For the supernatant, add absolute ethanol and dry the precipitate to obtain the water-extractable arabinoxylan. For the residue, add 0.25 mol/l NaOH and stir for 1 h at 60°C, then slowly add 30% H_2_O_2_ and hold for 4 h. Centrifuge the mixture, wash the residue once with 1 mol/l NaOH solution, and centrifuge again. Adjust the pH of the aqueous solution to 7 with 6 mol/l HCl, then add 4 volumes of absolute ethanol and dry the precipitate to obtain alkali-extractable arabinoxylan. RAX is the water-extractable arabinoxylan from rice bran and CAX is the alkali-extractable arabinoxylan from corn bran.

### 2.3. Bound ferulic acid depletion of arabinoxylan

Depletion of bound FA from AXs was performed as previously described with slight modifications (Snelders et al., 2013). FA was enzymatically hydrolyzed from AXs (5 g/100 ml deionized water) with a feruloyl esterase (230 μl/5 g AX) treatment at pH = 7 and 50°C for 24 h, boiled for 15 min to inactivate the feruloyl esterase. Then remove the free FA with Amberlite XAD-4 column, the solution was brought over the column with peristaltic pump and the run was collected and loaded on the column for another two times. Then the column was washed twice with deionized water, the wash fraction and the fraction of the reloaded solution fraction as AX solution, then absolute ethanol was added, and the precipitate was dried to obtain the ferulic acid depleted AX. RAX and CAX depleted the bound FA to obtain dRAX and dCAX, respectively.

### 2.4. Bound ferulic acid content of arabinoxylan

The bound FA content of AXs was measured as previously described with slight changes (Snelders et al., 2013). For determination of total FA, samples were hydrolyzed by addition of 2 mol/l NaOH and incubated for 2 h to release the ferulate group from the conjugate under N_2_ atmosphere. Then 6 mol/l HCl was added to adjust the pH to 2.0 and acidify the released ferulate group. The free FA is then extracted from the aqueous solution with three volumes of ethyl acetate three times. The pooled ethyl acetate extracts are then evaporated to dryness and resuspended in a known volume of methanol. For the determination of free FA, no saponification step occurred, and samples were analyzed from the acidification step. The amount of total and free ferulic acid is then quantified using high performance liquid chromatography (Agilent 1260, United States). The system combined with a UV detector at 320 nm and a Select C18 column (Silgreen GHAAL12S05, Greenherbs, China) at 20°C. The mobile phase contained solvent A (1.0% glacial acetic acid-water solution) and solvent B (acetonitrile) at a flow rate of 0.8 ml/min. 80% solvent A and 20% solvent B were eluted isocratically.

### 2.5. Monosaccharide and uronic acid compositions of arabinoxylan

The monosaccharide and uronic acid composition of AXs was analyzed by high-performance anion-exchange chromatography (ICS-6000, Thermo Scientific, United States) with pulse amperometric detection (Xie et al., 2013). The AX samples (20 mg) were hydrolyzed with 0.5 ml of 12 mol/l H_2_SO_4_ in a sealed glass tube and stirred for 30 min with a magnetic stirrer in an ice bath. Then, the H_2_SO_4_ was diluted to 2 mol/l for another 4 h of hydrolysis at 105°C. After the solution cooled to room temperature, the concentration of the mixture was diluted to about 100 μg/ml. The monosaccharide standards (including rhamnose, fructose, arabinose, xylose, galactose, mannose, and glucose) and the uronic acid standards (galacturonic acid and glucuronic acid) were dissolved in ultrapure water. The solution was filtered through a 0.22 μm syringe filter before injection into the system. For analysis, a CarboPac^™^ PA20 guard column (3 mm × 30 mm) and a CarboPacTM PA20 analytical column (3 mm × 150 mm) were connected in series. After injection of 25 μl of the sample, a quaternary gradient system consisting of ultrapure water (eluent A), 10 mmol/l NaOH solution (eluent B), 250 mmol/l NaOH (eluent C), and 500 mmol/l CH_3_COONa (eluent D) at 30°C was used to separate monosaccharide and uronic acid. UV detection was performed at 280 nm and the flow rate was 0.5 ml/min.

### 2.6. *In vitro* digestive pretreatment

Samples were subjected to simulated upper gastrointestinal tract digestion using a previous method with slight modifications (Lebet et al., 1998). The sample (2 g dry weight) was resuspended in 14 ml phosphate buffer saline (20 mM. pH = 6.9). The a-amylase solution (224 μl. 28.4 U/ml) was added and the mixture was then continuously stirred on a magnetic stirrer at 37°C for 10–20 min. After the pH was adjusted to 2.0, pepsin (34 μl, 51 U/ml) was added and incubated at 37°C for 30 min with continuous stirring. The pH was adjusted to 6.9, then trypsin (334 μl, 5 mg/ml) was added and incubated at 37°C for 90 min with constant stirring. After digestion, the suspension was boiled for 5 min to inactivate the enzymes. The cooled suspension was dialyzed against purified water using 3 kDa dialysis membranes for 3 days, changing the water every 6 h, and then freeze-dried for *in vitro* fermentation.

### 2.7. *In vitro* fecal fermentation

The *in vitro* fermentation was conducted under strictly anaerobic conditions and performed as per the method (Long et al., 2015). Fresh stool was collected from three healthy donors between the ages of 22 and 28 years who had no history of intestinal disease and had not been treated with antibiotics for at least 3 months before stool collection. This study was approved by the Ethics Committee of Nanchang University. All experiments were performed under guidelines and regulations of the IRB protocol. Informed consent was obtained from all donors. Fresh stool samples were collected from participants and immediately transferred to an anaerobic workstation (Bactron300-2, Shellab Bactron Company, United States) containing 5% H_2_, 5% CO_2_, and 90% N_2_. Three volumes of PBS medium (PBS medium was sterilized and deoxygenated, 1 l of PBS medium contains 8 g NaCl, 0.2 g KCL, 1.15 g Na_2_HPO_4_, 0.2 g KH_2_PO_4_, 0.5 g L-cysteine, 1 ml 1 mg/ml resazurin, and the rest is distilled water) were added to the samples and shaken until dispersion. The slurries were filtered through four layers of gauze cloth to remove visible particles. The fecal inoculum solution (1 ml) and 4 ml of PBS medium (containing 50 mg each of RAX, dRAX, CAX, and dCAX) were mixed. FOS was used as a positive control. The mixtures were incubated at 37°C. After 0, 6, 12, 24, and 48 h, gas production was measured with a syringe, and culture samples were collected and centrifuged at 13000 rpm for 5 min. The supernatants were stored at –80°C until analysis of SCFAs. The precipitates obtained after 24 h from all samples and at the beginning (0 h of Blank) were used for DNA extraction.

### 2.8. Short-chain fatty acids analysis

SCFAs (containing acetate, propionate, and butyrate) were determined as previously described with slight modifications (Zhao et al., 2006). 400 μl of the supernatant was added to a 2 ml centrifuge tube. 1 mol/l HCl (80 μl) and 10 mmol/l 2-ethylbutanoic acid (65 μl) were added to the supernatant and vortex shaken. Then, filtered through a 0.22 μm syringe filter, 1 μl was injected for gas chromatographic measurements (7080B, Agilent, United States) using a capillary column (HP-FFAP, 30 m × 0.25 mm × 0.25 μm, United States). The flow rates of N_2_, H_2_, and air were 1.5, 40, and 400 ml/min, respectively. The program initial temperature was 60°C, rose to 160°C at 10°C/min, maintained at 160°C for 1 min, increased to 200°C at 10°C/min, and held at 220°C for 5 min. The injector and detector temperatures were set at 200 and 240°C, respectively.

### 2.9. 16S rRNA sequencing and bioinformatics

Precipitates from the ferments were used for DNA extraction as previously described (Yu et al., 2021). DNA was extracted using the OMEGA Soil DNA Kit (D15625163 01; Omega Bio-Tek, Norcross, GA, United States). The concentration of extracted DNA was determined using a NanoDrop ND-1000 spectrophotometer (Thermo Fisher Scientific, Waltham, MA, United States), and purity was determined by agarose gel electrophoresis. Forward primer 338F (5′-ACTCCTACGGGAGGCAGCA-3′) and reverse primer 806R (5′-GGACTACHVGGGTWICTAAT-3′) were amplified at the V3–V4 regions of the bacterial 16S sRNA gene by PCR. Sample-specific 7-bp barcodes were inserted into the primers for multiplex sequencing. PCR amplicons were purified using VAHTS DNA Clean Beads (Vazyme, Nanjing, China) and quantified using the Quant-iT PicoGreen dsDNA Assay Kit (Invitrogen, Carlsbad, CA, United States). After individual quantification, all PCR amplicons were combined in equal amounts, and pair-end 2 × 250 bp sequencing was performed using the Illumina NovaSeq6000 system according to standard protocols at Shanghai Personal Biotechnology (Shanghai, China).

The obtained fastq sequences were further processed using the DADA2 pipeline with QIIME2 2019.4 (Callahan et al., 2016). Briefly, raw sequence data were demultiplexed using the demux plugin and primers were cut using the cutadapt plugin (Martin, 2011). Sequences were then quality filtered, denoised, merged, and chimeras removed using the DADA2 plugin (Callahan et al., 2016). Non-singleton amplicon sequence variants (ASVs) were aligned using mafft (Katoh et al., 2002) and used to generate a phylogeny using fasttree2 (Price et al., 2009). Alpha diversity metrics, including observed species and Shannon index, and beta diversity metrics were estimated using the diversity plugin, diluting samples to 35,582 sequences per sample. Taxonomy was assigned to ASVs using the classification Klearn naive Bayes taxonomy classifier in the Feature Classifier plugin (Bokulich et al., 2018) against the Greengenes database (v.13.8; DeSantis et al., 2006), which was set as the reference for taxonomic classification of each ASV. The original papers presented in this study are in the public domain. These data can be found at: https://doi.org/10.6084/m9.figshare.21695396.v1.

### 2.10. Statistical analysis

All experiments were performed in triplicate. Statistical differences between groups in the parameters of physicochemical properties, gas production, SCFAs production and α-diversity were analyzed using one-way analysis of variance (ANOVA) and Tukey’s *post hoc* test. The LEfSe method was performed at the ASVs level (using one-against-all comparisons, less strict) with LDA score large than 3 and assessed by Wilcoxon text. Data were analyzed using SPSS Statistics software (version 26.0, Ine. Chicago. IL, United States) and R 4.1.3. A significance level of *p* < 0.05 was set for all tests.

## 3. Results and discussion

### 3.1. Structural properties of the arabinoxylan

Two arabinoxylans were obtained from rice bran (RAX) and corn bran (CAX). The monomeric units of RAX and CAX were both predominantly xylose and arabinose (Table 1). The A/X ratio of RAX was 1.11, which was greater than that of CAX (A/X ratio 0.82). A higher A/X ratio was also found in arabinoxylans from rice bran compared to arabinoxylans from corn bran, suggesting that the structure of RAX is more branched and has complex side chains (Rose et al., 2010). RAX and CAX were then incubated with ferulic acid esterase to obtain the deferuloylated counterparts. The content of bound FA was reduced from 0.98 and 1.05 mg/g in RAX and CAX to 0.17 and 0.15 mg/g in dRAX and dCAX, respectively (Table 1).

**Table 1:** Bound ferulic acid content (mg/g AX) and monosaccharide compositions (%) of RAX, CAX, dRAX, and dCAX.

	Bound ferulic acid (mg/g AX)	Monosaccharide compositions (%)							A/X ratio
		Arabinose	Xylose	Galactose	Glucose	Fucose	Galacturonic acid	Glucuronic acid	
RAX	0.98 ± 0.05^a^	44.74 ± 2.29^a^	40.15 ± 1.52^b^	7.77 ± 3.82^a^	3.23 ± 0.44^a^	1.05 ± 0.08^a^	2.63 ± 0.09^a^	0.43 ± 0.26^b^	1.11 ± 0.02^a^
dRAX	0.17 ± 0.00^b^	44.84 ± 2.79^a^	40.45 ± 1.73^b^	7.42 ± 4.29^a^	3.21 ± 0.23^a^	0.95 ± 0.15^a^	2.77 ± 0.10^a^	0.35 ± 0.31^b^	1.11 ± 0.03^a^
CAX	1.05 ± 0.03^a^	36.83 ± 0.27^b^	44.92 ± 0.12^a^	10.94 ± 0.33^a^	2.65 ± 0.20^ab^	0.84 ± 0.03^a^	1.09 ± 0.06^b^	2.72 ± 0.18^a^	0.82 ± 0.00^b^
dCAX	0.15 ± 0.01^b^	36.67 ± 0.17^b^	45.56 ± 0.43^a^	10.93 ± 0.34^a^	2.16 ± 0.22^b^	0.99 ± 0.33^a^	1.04 ± 0.03^b^	2.65 ± 0.03^a^	0.80 ± 0.01^b^

Different superscript letters in the same column indicate significant differences (*p* < 0.05). RAX and dRAX stand for high and low bound ferulic acid rice bran arabinoxylan extracted with water, respectively. CAX and dCAX stand for high and low bound ferulic acid corn bran arabinoxylan extracted with 0.25 mol/l NaOH, respectively.

### 3.2. Effects of arabinoxylan on the gas production

**Figure 1:**
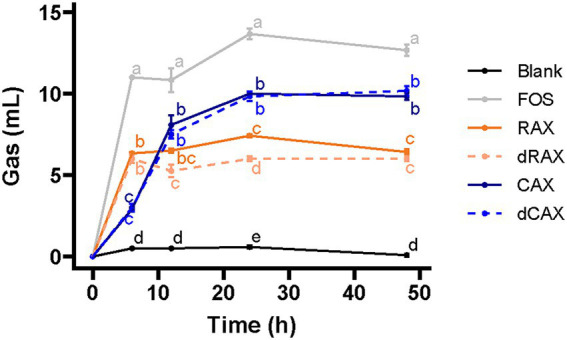
Gas production of RAX, CAX, dRAX, and dCAX compared to the Blank and FOS (positive control) by *in vitro* human fecal fermentation. FOS was used as a positive control. Blank did not contain any substrate. RAX and dRAX stand for high and low bound ferulic acid rice bran arabinoxylan extracted with water, respectively. CAX and dCAX stand for high and low bound ferulic acid corn bran arabinoxylan extracted with 0.25 mol/l NaOH, respectively.

Gas production is an important indicator of the fiber fermentation profile. The gas accumulation curve showed a two-phase fermentation pattern for all fibers (Figure 1). The slope of the first phase was largest for FOS and smallest for CAX. A flatter slope indicates slower gas accumulation and possibly slower fermentation. AXs were fermented more slowly than FOS. CAX and dCAX were fermented more slowly than RAX and dRAX. Removal of feruloylation did not affect the first phase of fermentation. RAX and dRAX both fermented rapidly within the first 6 h, then slowly until 48 h with little gas formation. After debranching in the first 6 h, the remaining AXs were poorly fermented and gas accumulation reached a plateau (Gu et al., 2021). The total gas accumulation of RAX and dRAX was lower than that of CAX and dCAX, which is related to the structural difference. Rice AXs which has more branches and complex side chains are more difficult to ferment than Corn AXs (Rumpagaporn et al., 2015). The decrease in gas accumulation after 12 h and 24 h in dRAX suggests that removal of feruloylation from RAX altered the fermentation pattern. Feruloylation was found to inhibit fermentation of AXOS. Specifically, removal of FA residues from feruloylated xylooligosaccharides makes them more amenable to further hydrolysis by xylanase and other degradative enzymes (Long et al., 2018). However, the effect of feruloylation on long chain AXs is still not clear. There is no difference between CAX and dCAX in gas accumulation suggesting that the inhibitory effect of FA depends on the arabinoxylan structure.

### 3.3. Effects of arabinoxylan on the SCFAs production

**Figure 2:**
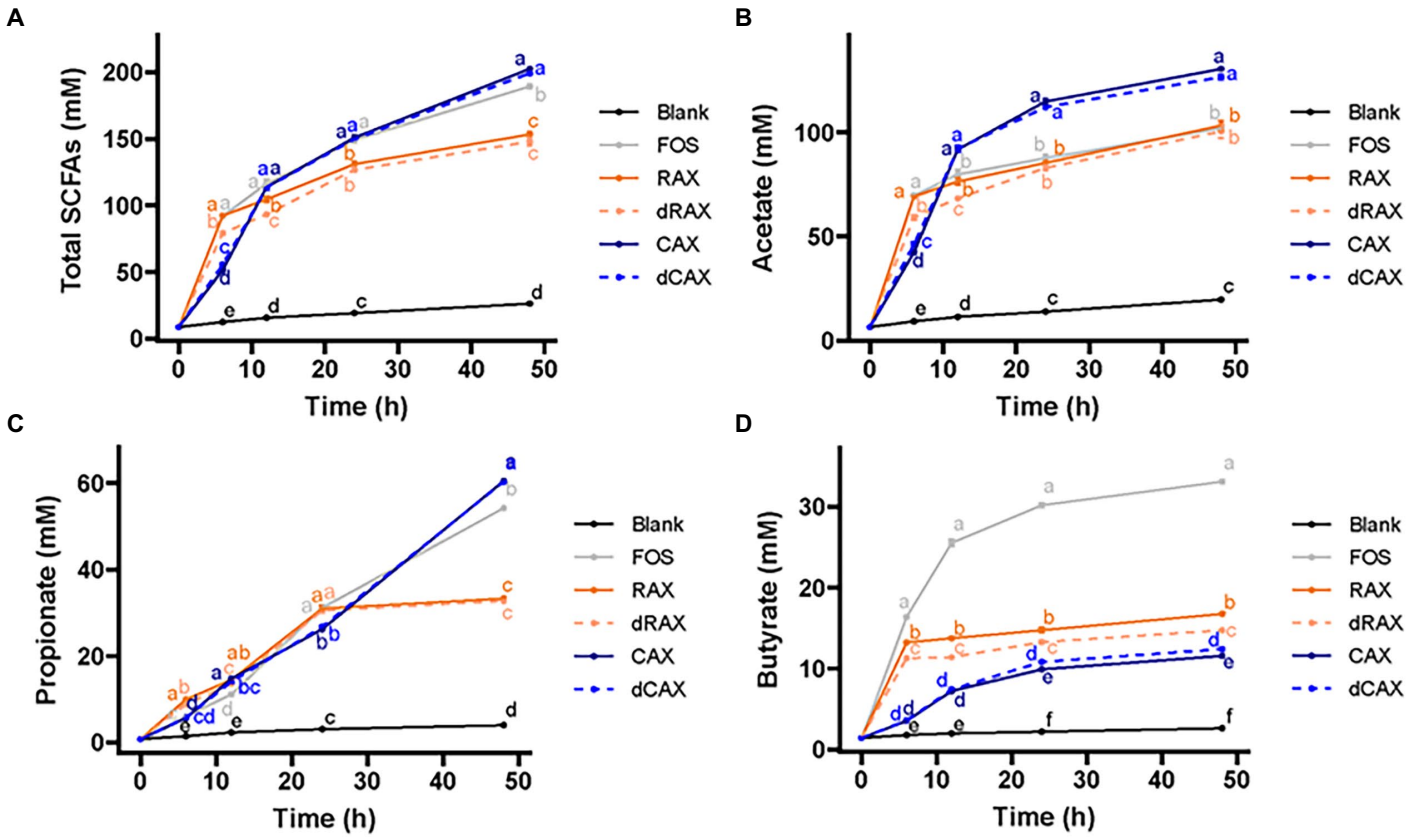
**(A)** Total SCFAs, **(B)** acetate, **(C)** propionate, and **(D)** butyrate production of each group by *in vitro* human fecal fermentation. Abbreviations are explained in Figure 1.

To better understand the effects of carbohydrate structure and feruloylation on the fermentation characteristics of AXs, the production of SCFAs during the 48 h fermentation period was evaluated. The overall production of SCFAs largely followed the trend of gas fermentation (Figure 2A). The major SCFAs in all AXs were acetate and propionate, followed by butyrate. Over the course of 48 h, acetate production showed a similar trend to the overall SCFA production (Figure 2B). Propionate production of AXs from rice bran increased significantly within 24 h and then reached a plateau, whereas propionate production of AXs from corn bran increased steadily within 48 h (Figure 2C). This is consistent with previous reports that AX induces an increase in propionate output (Chen et al., 2019; Nguyen et al., 2020). In the first 6 h, butyrate production exhibited a pattern comparable to that of total SCFA production. AXs from rice bran produced more butyrate than AXs from corn bran at all time points (Figure 2D). On the other hand, butyrate production from RAX was higher than that from dRAX and butyrate production from CAX was lower than that from dCAX. FA in the free from has been reported to accumulate butyrate by promoting the growth of butyrate-producing bacteria (Song et al., 2020). Similar to gas accumulation, the effect of bound FA on butyrate production was also not clear. Bound FA was found to inhibit fermentation on AXOS and did not affect butyrate concentration (Snelders et al., 2014). CAX with FA bound at 0.65 mg/g and 1.15 mg/g were similarly fermented and produced similar amounts of butyrate (Zhang et al., 2019). Here, the different effects of FA on butyrate were possibly due to the different structure of arabinoxylans. In summary, structural differences in carbohydrate structure were related to differences in gas and total SCFA production, and feruloylation was related to butyrate production.

### 3.4. Effects of arabinoxylan on the overall structure of gut microbiota

**Figure 3:**
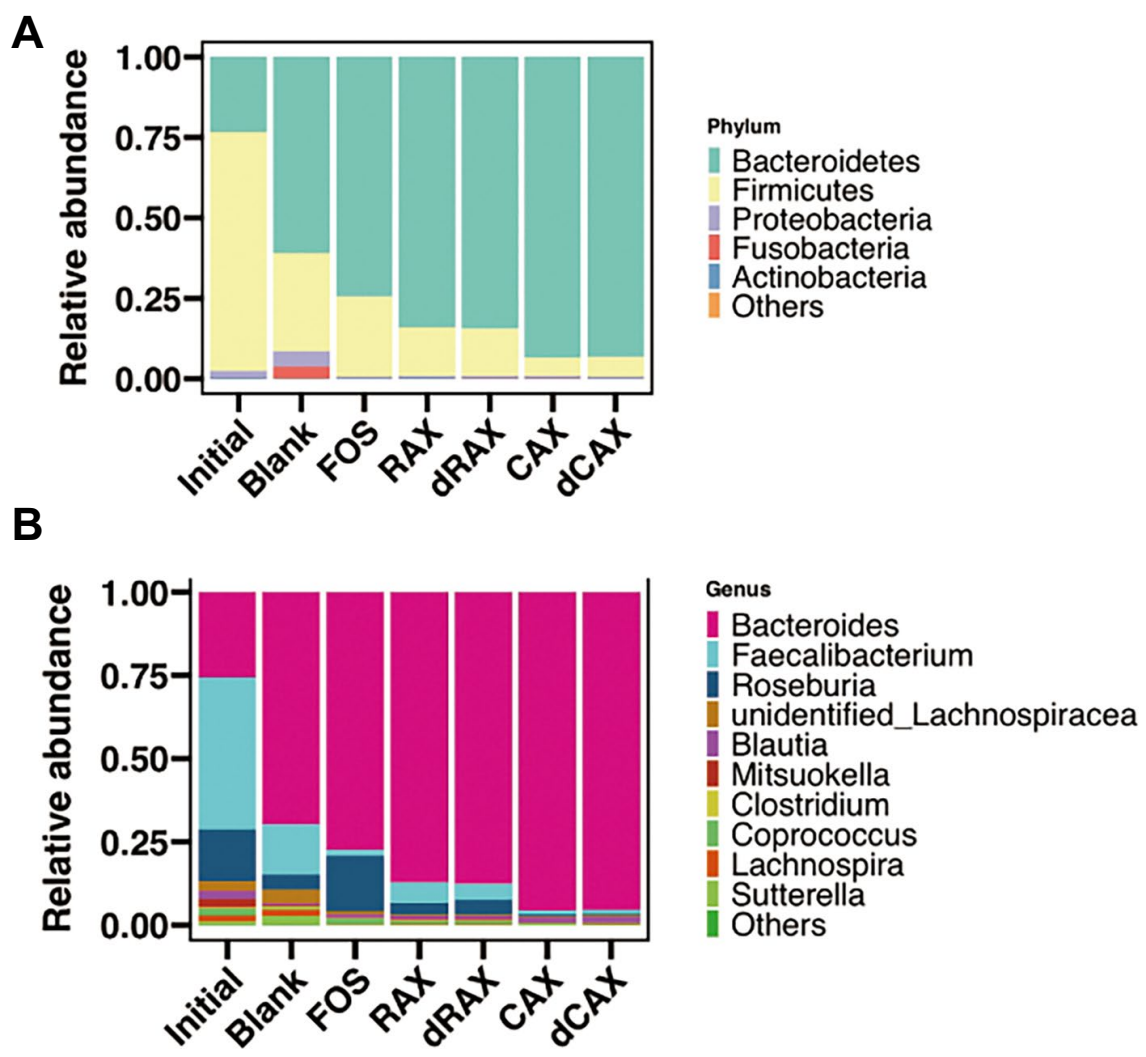
The relative abundance of the microbiota at the **(A)** phylum levels and the **(B)** genus levels of each group at 24 h *in vitro* fermentation. Abbreviations are explained in Figure 1.

Summary of taxa at the phylum level showed that the relative abundance of Bacteroidetes was increased during fermentation (Figure 3A). All AXs significantly increased the relative abundance of Bacteroidetes, but not the relative abundance of Proteobacteria and Fusobacterium. Another reason for the increase in relative abundance of Bacteroidetes may be the result of a decrease in Firmicutes. The genus *Faecalibacterium*, which belongs to the phylum Firmicutes, was predominant in the initial fecal sample and decreased significantly after 24 h of fermentation (Figure 3B). Compared with Blank and FOS, AXs promoted *Bacteroides*. The genera *Bacteroides* have been shown to possess the genetic and functional traits necessary for arabinoxylan utilization (Nguyen et al., 2020). The xylanolytic bacteria possessed polysaccharide utilization loci (PUL) that encode for the xylan utilization system (Zhang et al., 2014). *Prevotella* was another xylanolytic bacterium that was not presented due to interindividual differences (Fehlner-Peach et al., 2019). Unlike the selfish *Prevotella, Bacteroides supported* a complex metabolic web of cross-feeding interactions. Partial breakdown products were released by the action of xylan utilization system and shared with neighboring bacteria, which promoted the increase of alpha diversity (Grondin et al., 2017).

**Figure 4:**
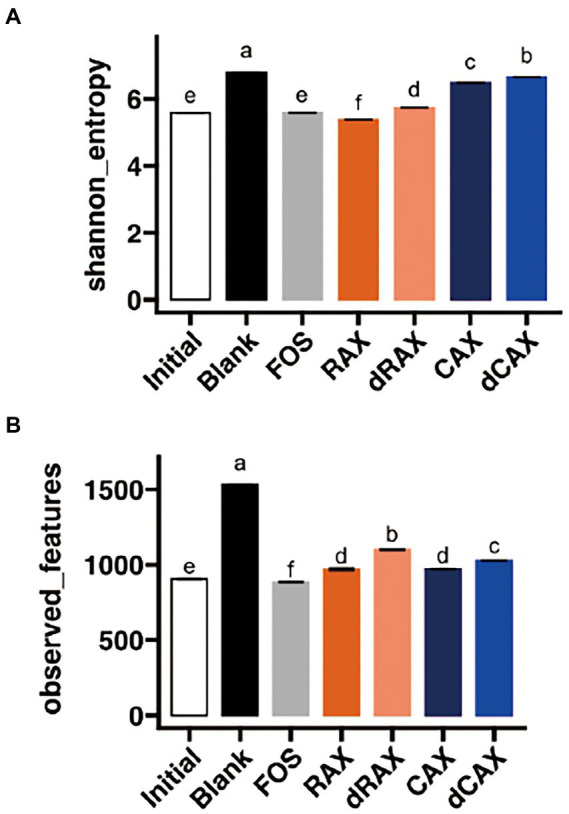
Alpha-diversity of the bacterial community as measured though **(A)** Shannon index and **(B)** observed features at 24 h *in vitro* fermentation. Abbreviations are explained in Figure 1.

The alpha diversity of the microbiota after 24 h fermentation was indicated by Shannon diversity, which is an indicator of both richness and evenness, and observed features, which is a direct indicator of ASV richness (Figure 4). After 24 h of fermentation, the Shannon diversity was significantly increased in the blank group (Figure 4A). This is due to the increase in observed features. It is unlikely that bacterial species increase in a closed ecosystem such as *in vitro* fermentation. The observed features were indicative of the coverage of the sampling depth (Figure 4B). The low abundance species that were initially not detected grew and became detectable in the Blank group. In comparison, these low abundance species remained present and undetectable in the fiber treatment groups. All AXs slightly increased the Shannon index and observed features compared to Initial. Long-chain AXs have been shown to restore the alpha diversity, which is decreased in high-fat diet fed mice (Neyrinck et al., 2011). Shannon index and observed features were lower in RAX than in CAX, possibly related to the difficulty of fermentation of RAX. Higher alpha diversity was observed in dRAX and dCAX than in RAX and CAX, respectively. Removal of FA is thought to slightly increase alpha diversity during fermentation. The *in vivo* study also showed that the binding of FA increased the Shannon diversity of the gut microbiota of XOS (Ou et al., 2016).

**Figure 5:**
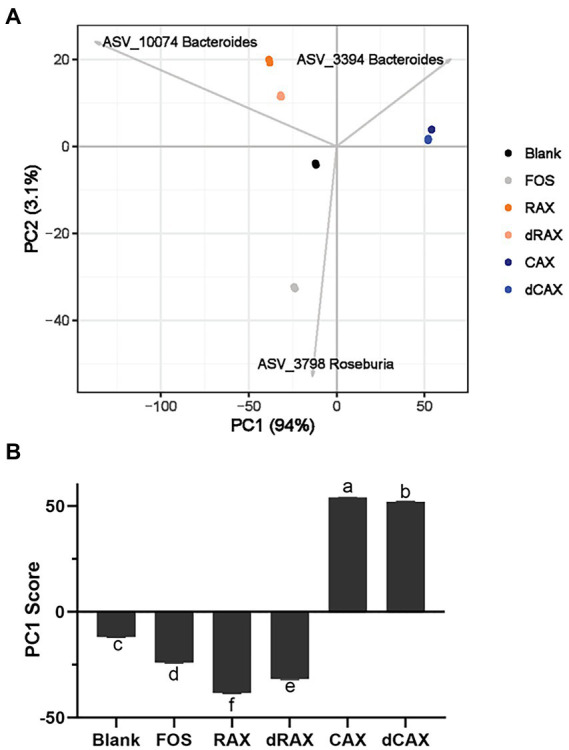
**(A)** The principal component analysis (PCA) of community structures and **(B)** the scores of the primary principle component (PC1) at 24 h *in vitro* fermentation with each group. Abbreviations are explained in Figure 1.

The PCA plot showed that the composition of the gut microbiota had shifted significantly by FOS and AXs after 24 h of fermentation (Figure 5A). On the PC1 axis, separation was observed between AXs from rice bran and corn bran, and the PC1 score was significantly different, explaining the different effect of AXs from two origins on the microbiota (Figure 5B). *Bacteroides* ASV_2294 has been associated with the degradation of maize AXs and *Bacteroides* ASV_10074 with the degradation of rice AXs. Although both belong to *Bacteroides*, these two *Bacteroides* may differ greatly in PULs. *B. thetaiotaomicron* and *B. ovatus* are the two most studied *Bacteroides* that have different glycan niches. Both *Bacteroides* harbor about 100 PULs, but almost few in common (Grondin et al., 2017). In addition, the removal of feruloylation also shifted the microbiota on PC1, suggesting feruloylation interference with the degradation of the AXs by *Bacteroides.*

### 3.5. Effects of arabinoxylan on the specific bacterial taxa as biomarkers in the microbiota

**Figure 6:**
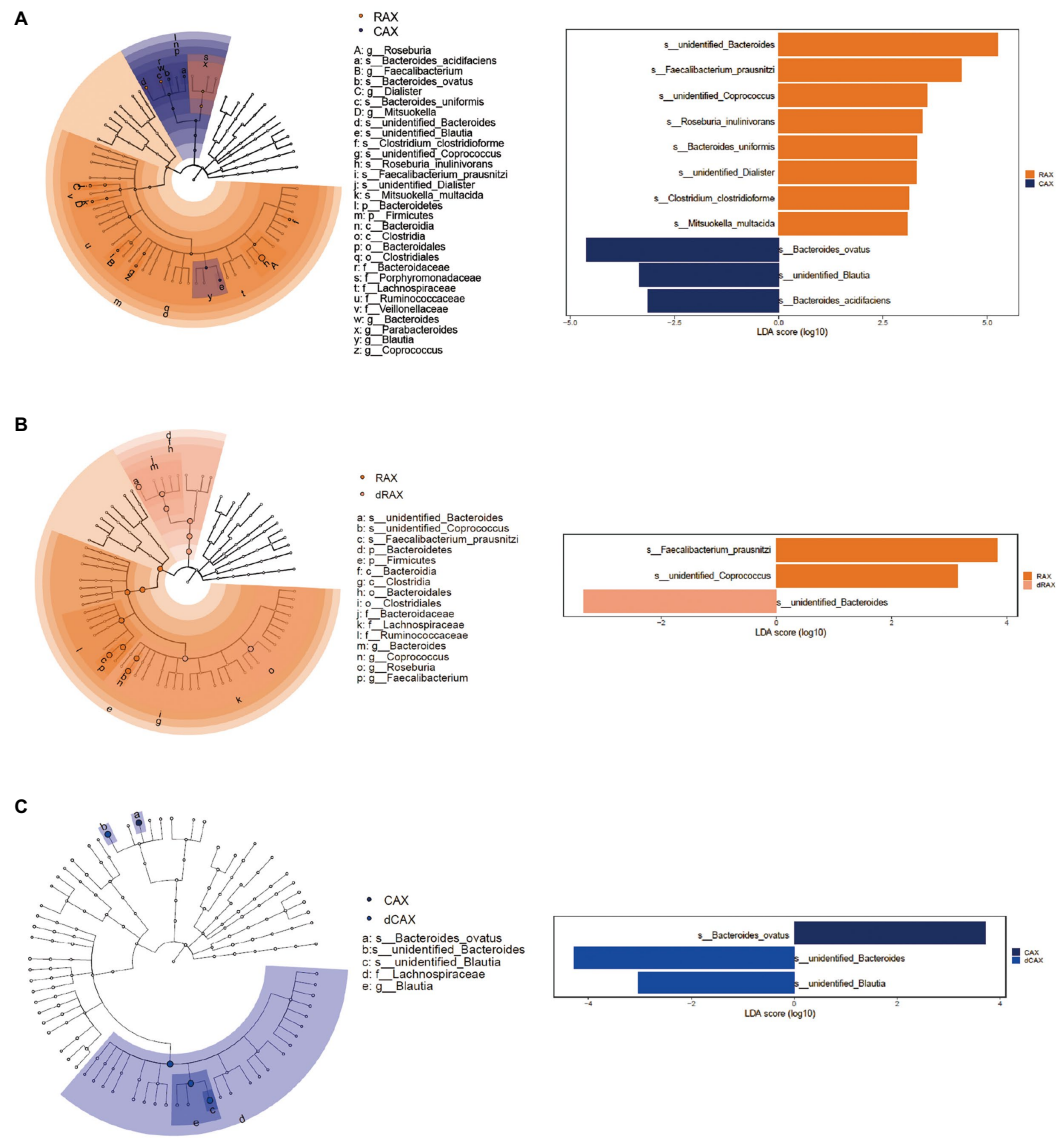
Most differentially enriched taxa between groups after 24 hours of *in vitro* fermentation, identified by LEfSe analysis **(A)** RAX and CAX, **(B)** RAX and dRAX, **(C)** CAX and dCAX. Each color represents a treatment group. The marker points in the taxonomic cladogram represent the difference at the different taxonomic levels between the compared groups. The corresponding taxa were labeled as taxa level followed by the names. The different species with a linear discriminant analysis (LDA) of more than 3 were listed with their respective LDA scores.

The bacteria responsible for the difference between groups were selected by LEfSe analysis with a linear discriminant analysis score greater than 3 (Figure 6). Comparing RAX and CAX, unidentified *Bacteroides*, *Faecalibacterium prausnitzi*, unidentified *Coprococcus*, *Roseburia inulinivorans*, *Bacteroides uniformis*, unidentified *Dialister*, *Clostridium clostridioforme*, and *Mitsuokella multacida* were more abundant in RAX, whereas *Bacteroides ovatus*, unidentified *Blautia*, and *Bacteroides acidifaciens* were more abundant in CAX. In agreement with PCA analysis, different species of *Bacteroides* were responsible for the utilization of RAX and CAX, respectively. In addition, butyrogenic bacteria, including *Faecalibacterium prausnitzi* and *Roseburia inulinivorans,* which are more abundant in RAX, explained the higher butyrate production of RAX than CAX (Figure 2D). *Roseburia* and *Blautia* are the main genera belong to the Lachnospiracea family. Genomic analysis of Lachnospiraceae revealed a considerable capacity to utilize diet derived polysaccharides including starch, inulin and AX. *Roseburia* species are able to degrade AXs through the activity of xylanase, α-L-arabinofuranosidase and β-xylosidase (Vacca et al., 2020).

Arabinoxylans are primarily degraded by *P*. *copri*, *B*. *longum*, and certain *Bacteroides* species. In addition to *P*. *copri*, which behaves “selfishly” and recycles AXs itself, *B*. *longum* and certain *Bacteroides* species released AXOS and xylan through the activity of endoxylanase, β-xylosidase, and α-arabinofuranosidase (Despres et al., 2016; Nguyen et al., 2020). It was shown that the complexity of AXs molecules strongly influenced the fermentation rate (Chen et al., 2017). *Bifidobacterium* spp. are known to degrade xylan, but which were not identified in this study, possibly due to interindividual differences. *Bifidobacterium longum* is a primary degrader of AXs, capable of cleaving the complex AX structure by AX-degrading glycosidase including β-xylosidase and α-arabinofuranosidase (Komeno et al., 2019). Other *Bifidobacterium* species did not possess the AX-degrading genes and were therefore unable to degrade long-chain AX themselves, but can degrade AXOS (Riviere et al., 2014).

Compared to dRAX, *Faecalibacterium prausnitzi* was promoted in RAX, which explained the higher butyrate production in RAX than in dRAX. It is suggested that the bound FA could promote the butyrate-producing bacterium and increase the butyrate production after fermentation of AX. However, comparing dCAX and CAX, the differences were related to saccharolytic species *B. ovatus,* unidentified *Bacteroides* and *unidentified Blautia*. *Bacteroides ovatus* possesses feruloyl esterase, which leads to a different degradation pattern of the xylanolytic reaction (Kmezik et al., 2020). With the decrease of propionated producing *B. ovatus*, *Blautia*, a butyrate-producing bacterium was increased in dCAX, which explained the increase of butyrate production in dCAX compared to CAX. Other ferulic acid esterase active bacteria such as lactic acid generating bacteria (*Limosilactobacillus*, *Lentilactobacillus*, *Bifidobacterium*, etc.) that were identified in this study possibly due to the microbiota composition of the fecal inoculum. It is suggested that feruloylation may interference with the xylanolytic pattern. And the impact of feruloylation differed depending on the AX structures.

Bound FA reduced the fermentability of AXOS because the feruloylated and diferuloylated arabinose substituents limited the access of xylanolytic enzymes to their target sites (Snelders et al., 2014). Compared to AXOS, AX is more complex and requires a larger repertoire of hydrolases to access and utilize the complex structure. AX usually embedded in the complex cell wall matrix with the presence of monomers and dimers of FA. Xylanase is required to dissolve part of the cell wall structure by forming short feruloylated xylooligosaccharides, and the presence of feruloylation hinders xylanase activity. Feruloyl esterases have been used to release FA and diFA from the complex cell wall and led to further degradation of AX (Oliveira et al., 2019). It was believed that the removal of FA residues from feruloylated xylooligosaccharides makes them again more accessible for further hydrolysis by xylanase and other degrading enzymes (Long et al., 2018). Similarly, when the long-chain AXs were cross-linked by FA, the xylan backbone was more resistant to degradation and the arabinose side chains were utilized first. The gel-like structure sterically hindered enzyme activity (Hopkins et al., 2003; Zhang et al., 2019). Compared to AXOS and AX matrix, the presence of bound FA only showed influence on a couple of bacteria for extracted AX polymers. For AX polymers that are released from the cell wall matrix, steric hinder induced by crosslink no longer existed. Compared to feruloylated AXOS, the amount of bound FA in CAX and RAX are relatively low. This also partially explained the only 6 bacteria species are affected by the bound FA.

## 4. Conclusion

In conclusion, AXs with different structure and feruloylation fermented differently. Fermentability was mainly determined by the carbohydrate backbone and its branches, including pH, gas production, and SCFAs profile. Although feruloylation had a minor effect on the fermentation pattern, it showed a significant effect on specific bacteria. Compared to RAX, removal of feruloylation significantly reduced the amount of *Faecalibacterium prausnitzi*, a known beneficial bacterium that promotes butyrate production. Compared to CAX, removal of feruloylation significantly reduced the amount of *Bacteroides ovatus*, a known propionate-producing bacterium that degrades complex carbohydrates, and increased the amount of *Blautia*, a butyrate-producing bacterium. The effects of feruloylation on the fermentation pattern and the resulted microbiota shift of AX varied depending on the carbohydrate structure.

## Data availability statement

The original contributions presented in the study are included in the article/supplementary material, further inquiries can be directed to the corresponding authors.

## Ethics statement

The studies involving human participants were reviewed and approved by Ethics Committee of Nanchang University. The patients/participants provided their written informed consent to participate in this study.

## Author contributions

ZL: conceptualization and methodology. HZ: data curation, writing-original draft preparation, and investigation. LH: validation. YH: formal analysis. YC: visualization. TL: resources. SX: software. XZ: reviewing and editing. SL: supervision and investigation. CL: supervision and salidation. TC: conceptualization and writing-reviewing and editing. All authors contributed to the article and approved the submitted version.

## Funding

This study was financially supported by the National Natural Science Foundation of China (Nos. 31901703, 32160530, and 32072257), China Postdoctoral Science Foundation (2020M671974), the Natural Science Foundation of Jiangxi Province (No. 20202BAB215015), Central Government Guide Local Special Fund Project for Scientific and Technological Development of Jiangxi Province (20212ZDD02008), and Jiangxi Research Project Funding for Selected Postdoc (2020KY07).

## Conflict of interest

The authors declare that the research was conducted in the absence of any commercial or financial relationships that could be construed as a potential conflict of interest.

## Publisher’s note

All claims expressed in this article are solely those of the authors and do not necessarily represent those of their affiliated organizations, or those of the publisher, the editors and the reviewers. Any product that may be evaluated in this article, or claim that may be made by its manufacturer, is not guaranteed or endorsed by the publisher.
